# Dextromethorphan Mediated Bitter Taste Receptor Activation in the Pulmonary Circuit Causes Vasoconstriction

**DOI:** 10.1371/journal.pone.0110373

**Published:** 2014-10-23

**Authors:** Jasbir D. Upadhyaya, Nisha Singh, Anurag S. Sikarwar, Raja Chakraborty, Sai P. Pydi, Rajinder P. Bhullar, Shyamala Dakshinamurti, Prashen Chelikani

**Affiliations:** 1 Department of Oral Biology, University of Manitoba, Winnipeg, MB, Canada; 2 Departments of Pediatrics, Physiology, University of Manitoba, Winnipeg, MB, Canada; 3 Manitoba Institute of Child Health, Winnipeg, MB, Canada; University of North Dakota, United States of America

## Abstract

Activation of bitter taste receptors (T2Rs) in human airway smooth muscle cells leads to muscle relaxation and bronchodilation. This finding led to our hypothesis that T2Rs are expressed in human pulmonary artery smooth muscle cells and might be involved in regulating the vascular tone. RT-PCR was performed to reveal the expression of T2Rs in human pulmonary artery smooth muscle cells. Of the 25 T2Rs, 21 were expressed in these cells. Functional characterization was done by calcium imaging after stimulating the cells with different bitter agonists. Increased calcium responses were observed with most of the agonists, the largest increase seen for dextromethorphan. Previously in site-directed mutational studies, we have characterized the response of T2R1 to dextromethorphan, therefore, T2R1 was selected for further analysis in this study. Knockdown with T2R1 specific shRNA decreased mRNA levels, protein levels and dextromethorphan-induced calcium responses in pulmonary artery smooth muscle cells by up to 50%. To analyze if T2Rs are involved in regulating the pulmonary vascular tone, *ex vivo* studies using pulmonary arterial and airway rings were pursued. Myographic studies using porcine pulmonary arterial and airway rings showed that stimulation with dextromethorphan led to contraction of the pulmonary arterial and relaxation of the airway rings. This study shows that dextromethorphan, acting through T2R1, causes vasoconstrictor responses in the pulmonary circuit and relaxation in the airways.

## Introduction

Taste perception fulfills an essential role in evaluating the quality and nutritional value of food prior to ingestion. Humans can taste many compounds but are able to distinguish between five basic tastes, which are bitter, sweet, umami, salt and sour. The signal transduction for sweet, umami and bitter tastes is mediated through G protein-coupled receptors (GPCRs) [1], [2]. Bitter taste provides a defense mechanism against the ingestion of toxic substances. In humans, bitter taste is sensed by a family of 25 GPCRs, referred to as T2Rs, which are localized in clusters on chromosomes 5p15, 7q31 and 12p13 [3], [4]. The human T2Rs are intronless genes. The ligands that activate T2Rs have diverse chemical structures and include natural alkaloids, such as quinine, nicotine, and synthetic compounds such as dextromethorphan (DXM).

Recent studies indicate that in addition to their expression in gustatory system, T2Rs are expressed in extra-oral regions, such as the respiratory circuit [5]–[8], gastrointestinal tissues [9], reproductive tissues [10], mesenchymal stromal and vascular smooth muscle [11], and the brain [12]. The taste receptor signaling cascade seems to be remarkably conserved among tissues, however, T2Rs elicit very diverse effects in different tissues, thus suggesting that they have additional functions apart from sensing taste [13]. In gastrointestinal endocrine cells, T2Rs upon activation with bitter compounds, secrete the peptide hormones ghrelin and glucagon-like peptide-1 and thus, play a role in the modulation of glucose homeostasis [14]. In human airway epithelia, bitter compounds stimulate the ciliary activity to hasten the elimination of harmful substances and initiate protective airway reflexes [6]. Activation of T2Rs in airway smooth muscle cells (ASMCs) leads to muscle relaxation and bronchodilation, that is three fold greater than that elicited by currently used beta-adrenergic receptor agonists [7]. Previously the expression of TAS2R46 was reported in human aortic smooth muscle cells and rats injected with denatonium showed a significant drop in their blood pressure [11]. However, the presence of all the 25 human T2Rs in other vascular tissues like the pulmonary artery smooth muscle (PASM) has not been established.

In the present study, we characterized the expression of T2Rs in pulmonary artery smooth muscle cells (PASMCs) as well as the effects of bitter agonist DXM on pulmonary artery. Using reverse-transcriptase (RT)-PCR, we show the expression of multiple T2R transcripts in hPASMCs. Functional studies on these cells indicated an increase in intracellular calcium levels after the application of many natural and synthetic bitter tasting compounds, suggesting that T2Rs in hPASMCs are functional. Since we have previously characterized the response of T2R1 to DXM [15], this receptor was selected for further analysis in the study. Knockdown of T2R1 by transfection of hPASMCs with T2R1 specific shRNA reduced the mRNA levels by 50±12%, protein levels by 54±2%, and DXM-induced intracellular calcium levels by up to 50%. Myograph studies using porcine pulmonary arterial and airway rings showed that stimulation with DXM led to contraction of the pulmonary arterial rings and relaxation of the airway rings. Activation of T2Rs expressed in airways was recently shown to cause bronchodilation [7]. Taken together, our novel findings suggest that in the pulmonary circuit DXM acts as a vasoconstrictor, and shows that DXM mediated activation of T2R1 in pulmonary tissues leads to vasoconstrictor responses.

## Materials and Methods

### Materials

Dextromethorphan hydrobromide, chloroquine, denatonium benzoate, quinine hydrochloride, phenylthiocarbamide (PTC), 6-n-propylthiouracil (PROP), sodium thiocyanate, salicin, nicotine, yohimbine, colchicine, thiamine, caffeine, chloramphenicol and picrotoxinin were purchased from Sigma. Fluo-4 NW calcium assay kit was purchased from Invitrogen. hPASMCs and hASMCs were either purchased from ATCC or were a kind gift from Dr. Andrew Halayko, Dept. of Physiology, University of Manitoba. Cell culture media and hPASMC growth kit were purchased from Cedarlane, Canada. Nucleofection kit for smooth muscle cell transfection was purchased from Lonza. Polyclonal antibodies specific for T2R1, T2R38 and normal IgG specific rabbit antisera were obtained from Abcam (Cambridge, MA, USA) and Santa Cruz Biotechnology (Dallas, TX, USA) respectively [6], [7]. Phospho-MLC-Ser19 monoclonal antibody and MLC polyclonal antibody were purchased from Cell Signaling technology (Danvers, MA, USA). DyNazyme hot start was purchased from Thermo Fisher Scientific (Toronto, ON, Canada). The shRNA specific for T2R1 was purchased from Qiagen (Toronto, ON, Canada).

### Animals

Newborn piglets (<24 hours age) were obtained from a pathogen-free farm supplier on the day of experiment. Animals were euthanized with a lethal dose of pentobarbital (480 mg/kg intraperitoneal). Heart and lungs were removed en bloc into cold Krebs-Henseleit buffer. This protocol is approved by the University of Manitoba, per Canadian Council on Animal Care (Permit Number: 14-008).

### RNA preparation and Reverse Transcriptase (RT)-PCR

The HUGO gene nomenclature of TAS2R is used wherever the gene is mentioned. The 25 hTAS2R PCR primers were designed towards the available human sequences from NCBI Genebank using the OligoPerfect software from Invitrogen (Burlington, ON, Canada). The details of the primer sequences are given in **Table 1**. Total RNA from untransfected hPASMCs, hASMCs and from T2R1 specific shRNA and scrambled shRNA transfected cells was isolated according to manufacturer's instructions using the RNeasy Mini kit (Qiagen, Canada). The concentration and purity of the RNA was determined using a Nanodrop 2000 (Thermo Scientific, Canada). A small amount of total RNA was reverse transcribed into cDNA using SSIII RT (superscript III reverse transcriptase, Invitrogen, Canada). RT-PCR was performed according to previously published methods [12].

**Table 1:** DNA primer sequences for human bitter taste receptors (TAS2Rs)*.

Bitter receptor (TAS2R)	Gene Accession ID	Gene size (bp)	Primer Sequence (5′-3′)	Expected amplicon size (bp)
TAS2R1	NM_019599	1355	Forward-TGTGGTGGTGAATGGCATTG, Reverse- CAGCACTTACTGTGGAGGAGGAAC	813
TAS2R3	NM_016943	1101	Forward-ACACATGATTCAGGGATAATAATGCAAA, Reverse- TTAGCCATCTTGGTTTTTGGTAGGAAATT	575
TAS2R4	NM_016944	900	Forward-TACAGTGGTCAATTGCAAAACTTGG, Reverse- AATGTCCTGGAGAGTAAAGGGTGG	749
TAS2R5	NM_018980	1150	Forward-TGGTCCTCATATAACCTCATTATCCTGG, Reverse- CTGCCATGAGTGTCTCCCA	667
TAS2R7	NM_023919	1096	TGTTTTATATTGGTGCTATATCCAGATGTCTATGC, GGATAAATGAATGACTTGAGGGGTAGATTAGAG	658
TAS2R8	NM_023918	930	Forward-TTGATATGGTGGTGCACTGG, Reverse- GTGAGTGACCCAAGGGGTAG	471
TAS2R9	NM_023917	1075	Forward-TGAATTGACCATAGGGATTTGGG, Reverse- ATAATTAGAATGAATGAATGGCTTGATGG	807
TAS2R10	NM_023921	924	Forward-GACTTGTAAACTGCATTGACTGTGCC, Reverse- AAAGAGGCTTGCTTTAGCTTGCTG	784
TAS2R13	NM_023920	1637	Forward-GGGTCAGTAAAAGAGAGCTGTCCTC, Reverse- ATCAGAAGAAAGGAGTGGCTTGAAG	742
TAS2R14	NM_023922	954	Forward-GCTTTGGCAATCTCTCGAATTAGC, Reverse-CTCTAAATTCTTTGTGACCTGAGGGC	796
TAS2R16	NM_016945	996	For-CCTGGGAATTTTTTAATATCCTTACATTCTGGT, Reverse-GAAGCGCGCTTTCATGCTT	419
TAS2R38	NM_176817	1143	Forward-ACAGTGATTGTGTGCTGCTG, Reverse- GCTCTCCTCAACTTGGCATT	766
TAS2R39	NM_176881	1017	Forward-TGTCGCCATTTCTCATCACCTTA, Reverse- ATTGAGTGGCTGGCAGGGTAG	841
TAS2R40	NM_176882	972	Forward-AGAGTGCATCACTGGCATCCTT, Reverse- GAGGATGAGAAAGTAGCTGGTGGC	685
TAS2R41	NM_176883	924	Forward-GGTTGCTGCCCTTGGATATGA, Reverse- TGAAGATGAGGATGAAGGGATGG	738
TAS2R42	NM_181429	945	Forward-ATGGCCACCGAATTGGACA, Reverse- GCTTGCTGTTTCCCAGAATGAG	871
TAS2R43	NM_176884	1027	Forward-GGTCTCCAGAGTTGGTTTGC, Reverse- TCTTGTTTCCCCAAATCAGG	698
TAS2R44	NM_176885	1021	Forward-CATTGGTAAATTCCATTGAGC, Reverse- GATATCATTATGGACAGAAAGTAAAC	661
TAS2R45	NM_176886	900	Forward-CTCCTTTGCTGACCAAATTGTC, Reverse- GAACGGGTGGGCTGAAGAAC	709
TAS2R46	NM_176887	930	Forward-GAGTTGAATCCAGCTTTTAAC, Reverse- ATAGCTGAATGCAATAGCTTC	606
TAS2R47	NM_001097643	960	Forward-GGTGTTATTACTACATTGGTATGCAACTC, Reverse- AAGACAGGTTGCTTTTCCAGC	603
TAS2R48	NM_176888	900	Forward-GGTTTACTCTGGGTCATGTTATTC, Reverse- TTTGCTCTGCTGTGTCCTAAG	606
TAS2R49	NM_176889	1914	Forward-GCACTGATAAATTTCATTGCCTGG, Reverse- TTGTTCCCCCAAATCAGAATGAAT	770
TAS2R50	NM_176890	1000	Forward-ATGTGGCTTGCTGCTAACCT, Reverse- CAGCCTTGCTAACCATGACA	514
TAS2R60	NM_177437	957	Forward-CAGGCAATGGCTTCATCACTG, Reverse- TCCCACACCCAGAATTTAAAGTCC	748
GNAT3	NM_001102386	1065	Forward-GTGGCATGACACCTCAACTG, Reverse- GGCCCAGTGTATTCTGGAAA	529
GNAT1	NM_144499	3617	Forward-AGGGAATATCCCTCCCACAC, Reverse- CCAAGAAAGGACAGCTGGAG	843
GAPDH	NM_002046	1310	Forward-TGTGAGGAGGGGAGATTCAG, Reverse- ACCCAGAAGACTGTGGATGG	572

(*The HUGO gene nomenclature of TAS2R is used wherever the gene is mentioned).

### shRNA knockdown, hPASMC transfection by electroporation method, calcium mobilization, and Western blot experiments

hPASMCs (∼1×10^5^ cells) were suspended in 100 µl nucleofector solution. Following this, 2 µg of T2R1 shRNA, scrambled shRNA and 100 µl nucleofector solution were combined in separate tubes. A cell specific type nucleofector program was selected and applied to the cell/DNA mixture. The cells were taken out and resuspended in 500 µl prewarmed cell culture media and seeded in 6 well plate for further quantitative PCR (qPCR) and calcium mobilization experiments. Receptor activation was determined in untransfected and/or after 48 h of transfection as described before [15]. Changes in intracellular calcium were measured in terms of relative fluorescence units (RFU) after application of assay buffer alone (control) or 15 bitter compounds using a FlexStation 3 microplate reader (Molecular Devices, CA). Representative calcium traces are shown in Figure S1 in File S1.

For Western blot analysis, following 48 h of transfection with T2R1 specific shRNA or scrambled shRNA the cells were lysed using lysis buffer (Pierce Scientific, Canada) containing protease inhibitors. Cell lysates were separated by 12% SDS-PAGE and transferred to nitrocellulose membrane and subjected to immunoblotting using polyclonal antibody against T2R1 and β-actin (Sigma). The blot was visualized using ECL detection reagents (ThermoFisher Scientific, Canada).

### Quantitative (q) PCR

Total RNA was extracted from hPASMCs transfected with T2R1 specific shRNA or scrambled shRNA using the RNeasy mini kit (Qiagen). cDNA was synthesized using SSIII RT and qPCR was performed as described before [12]. The short-length primer sequence used for detecting hTAS2R1 is as follows: forward: GTCCGTCACCCACTCTTCAT; reverse: GGGACCATAAACCCTGCATA. Same conditions were followed for the quantitative analysis of T2R1 in hPASMCs and hASMCs. A Bio-Rad MJ mini opticon real time PCR detection system was used for these experiments.

### Immunofluorescence analysis in hPASMCs cells

hPASMCs, grown on coverslips, were processed for immunofluorescence analysis as described previously [15]. The cells were treated with primary antibodies for T2R1, T2R38 and the isotype-specific IgG sera (diluted in blocking solution at 1∶300) for 1 h at room temperature and visualized with Alexa fluorophores. Representative cells were selected and visualized using Olympus BX81 microscope for the localization of indicated proteins.

### Isolation, culture and qPCR of porcine PASMCs and ASMCs

4^th^–6^th^ generation pulmonary arteries and airways were dissected from newborn piglet (<24 hrs old) into ice cold Ca^2+^free Krebs-Henseleit buffer containing (in mM) 25 NaHCO_3_, 112.6 NaCl, 4.7 KCl, 1.38 NaH_2_PO_4_, 2.46 MgSO_4_.7H_2_O, 5.56 Dextrose; pH 7.4). The arteries and airways were allowed to recover in cold HEPES-buffered saline solution (HBS); composed of (in mM) 130 NaCl, 5 KCl, 1.2 MgCl_2_, 1.5 CaCl_2_, 10 HEPES, 10 glucose; pH 7.4. Pulmonary arterial and airway smooth muscle cells were obtained using a dispersed cell culture method as described previously [16]. Briefly, pulmonary arteries and airways were washed twice with a 20 µM CaCl_2_ (reduced-Ca^2+^) HBS solution, finely minced, then transferred to a digestion medium containing reduced-Ca^2+^ HBS, type I collagenase (1,750 U/mL), dithiothreitol (1 mM), bovine serum albumin (2 mg/ml), and papain (9.5 U/mL) for 15 min at 37°C with gentle agitation. The dispersed cells were collected by centrifugation at 1,200 rpm for 5 min, washed in Ca^2+^-free HBS to remove digestion solution, then resuspended in Ham's F-12 medium supplemented with 10% fetal bovine serum, 1% penicillin and 1% streptomycin and seeded at a density of 4.4×10^4^ cells/cm^2^. Experiments were performed when cells reached 80% confluence.

Total RNA was extracted from piglet PASMCs and ASMCs using the RNeasy mini kit (Qiagen). Isolated RNA was treated with DNase I and then used for cDNA synthesis with SSIII RT, dNTPs, OligodT primer and first strand buffer (Invitrogen). Reverse transcription was carried out as described previously. The primer sequences for piglet T2R1 were the same as used in the study by Colombo *et al.* [17]. Reaction mixtures with a final volume of 20 µl consisted of 2 µl reverse transcribed cDNA, 5 pmol primers, 1x SYBR green containing dNTP mix and Taq polymerase. The reaction consisted of the following steps; an initial denaturation step of 1 min at 95°C then 50 cycles of 94°C for 30 sec, annealing at 60°C for 30 sec and extension at 72°C for 30 sec and a final extension at 72°C for 2 min. This was followed by melt curve analysis from 72°C to 95°C at every 1°C increase in temperature for about 1 sec for 23 cycles. Melt curve analysis confirmed the presence of a single PCR product in each reaction. These experiments were pursued on a Bio-Rad MJ mini opticon real time PCR detection system.

### Isometric Myography

Pulmonary arteries and airways (100–300 µm luminal diameter, 4^th^–6^th^ generation) from day 0 piglet were gently cut into 2–3 mm rings. The diameter was determined by using micrometer slide and an eyepiece reticule. Rings were optimally equilibrated at a median resting tension of 1.5 mN on mounting pins attached to a force transducer on a multi-chamber isometric wire myograph (*Danish Myo Systems*) in Krebs-Henseleit buffer at 37°C, continuously bubbled with a 95% O_2_, 5% CO_2_ gas mixture to give a pH of 7.40 to 7.45. Pulmonary artery ring normalization was modified from Mulvany and Halpern [18] for isometric myography of small resistance arteries, by stepwise addition of resting tension until a small accrual of passive tension was observed; resting tension was then decreased by one step below that level, to set the vessel at optimal resting tension for isometric study [19]. After equilibration, maximum active tension to KCl stimulation was determined for each ring. Thromboxane A2 receptor (TP) mimetic U46619 (30 nM), a potent vasoconstrictor for vascular smooth muscle, was used for pre-contracting the arterial rings. In selected rings, endothelium was removed by gentle rotation of the ring on fine steel rod in cold Krebs-Henseleit buffer; rings were considered endothelium-denuded when artery rings pre-contracted by challenge with thromboxane receptor (TP) mimetic U46619 (10^−6^ M), exhibited an acetylcholine (10^−5^ M) mediated relaxation of no greater than 5% of pre-existing tone, compared to control rings. Isometric tension in response to agonists was recorded by computer using Powerlab data collection and Chart 5 software; contraction was expressed relative to maximal KCl-induced force. A minimum of n = 15 rings from 5 piglets were used for each experiment.

### Detection of superoxide by Dihydroxyethidium (DHE) fluorescence method

Superoxide was measured in hPASMCs by DHE fluorescence method. hPASMCs (∼1×10^5^ cells) were cultured in serum deprived condition for 72 h in 5% CO_2_ and 21% O_2_. The cells were then treated with 0.5 mM DXM for 4 h. DHE stock (31.7 µM) was prepared in 10 mg/ml DMSO and then diluted in PBS. Cells were washed twice in PBS and then loaded with DHE for 45 min at 37 °C. The excess dye was washed off, and the intracellular fluorescence intensity was detected using a FlexStation 3 microplate reader with the following settings, excitation at 500 nm and emission at 610 nm [20], [21].

### Measurement of inositol-1,4,5-trisphosphate (IP_3_) and analysis of expression of Myosin Light Chain (MLC) and phosphorylation of MLC (phospho MLC)

IP_3_ assays were carried out in human and porcine PASMCs and ASMCs using a commercially available IP_3_ assay kit (HitHunter IP_3_ fluorescence polarization [FP] assay, DiscoveRx, Fremont, CA) according to the instructions supplied by the manufacturer and as described previously [22]. For MLC and phospho MLC experiments, porcine PASMCs and ASMCs were treated with 500 µM DXM or 1 µM U46619, or buffer alone for 20 minutes and cells were lysed with ice-cold lysis buffer (Pierce Scientific) containing protease inhibitors. The cell lysate was then separated by 10% SDS-PAGE and Western blot was performed. Membranes were incubated with primary antibody, either a 1∶500 dilution of phospho-MLC-Ser19 monoclonal antibody or 1∶200 dilution of MLC polyclonal antibody, overnight at 4°C. Blots were washed and then probed with a 1∶4000 dilution of peroxidase conjugated secondary antibody and visualized using ECL detection reagents.

### Statistical Analysis

All data are presented as mean ± SEM. Statistical analysis was performed using one-way ANOVA with Tukeys or Dunnetts *post hoc* test or student *t*-test wherever applicable. GraphPad Prism (version 4) was used for the analysis.

## Results

### Expression of TAS2R transcripts and functional characterization

**Figure 1:**
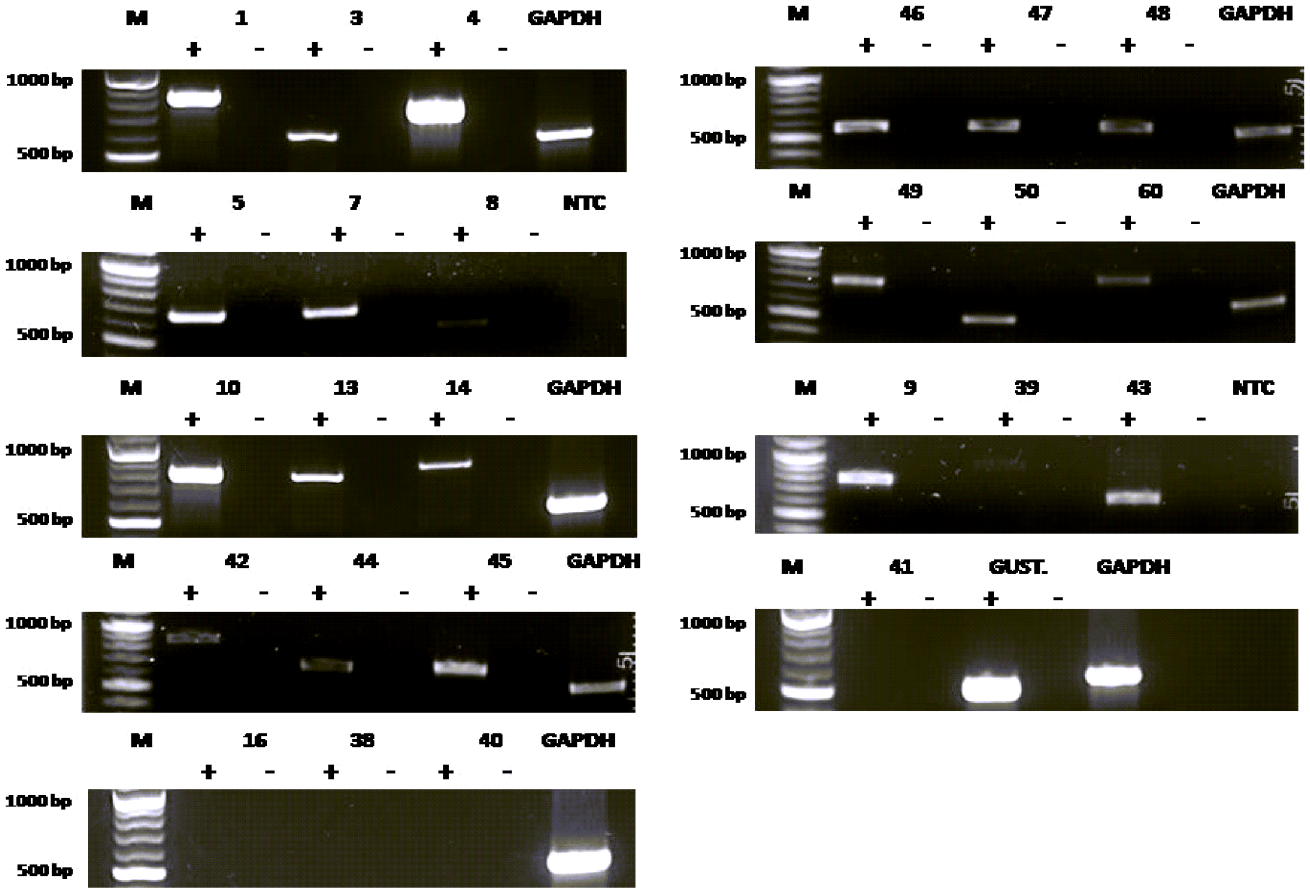
Reverse transcriptase (RT)-PCR analysis of the expression of bitter taste receptors (TAS2Rs) in hPASMCs. Agarose gel electrophoresis (1%) analysis of the RT-PCR products showed that 21 T2Rs were expressed in hPASMCs. GAPDH was used as an internal control for the PCR reactions. + and – represent the addition and omittance of reverse transcriptase in the reaction respectively. NTC represents a no template control in which the cDNA template was omitted. M represents 100 bp molecular weight standard (NEB). All transcripts were observed at the expected amplicon size. Each agarose gel electrophoresis is representative of 4–5 independent experiments.

**Figure 2:**
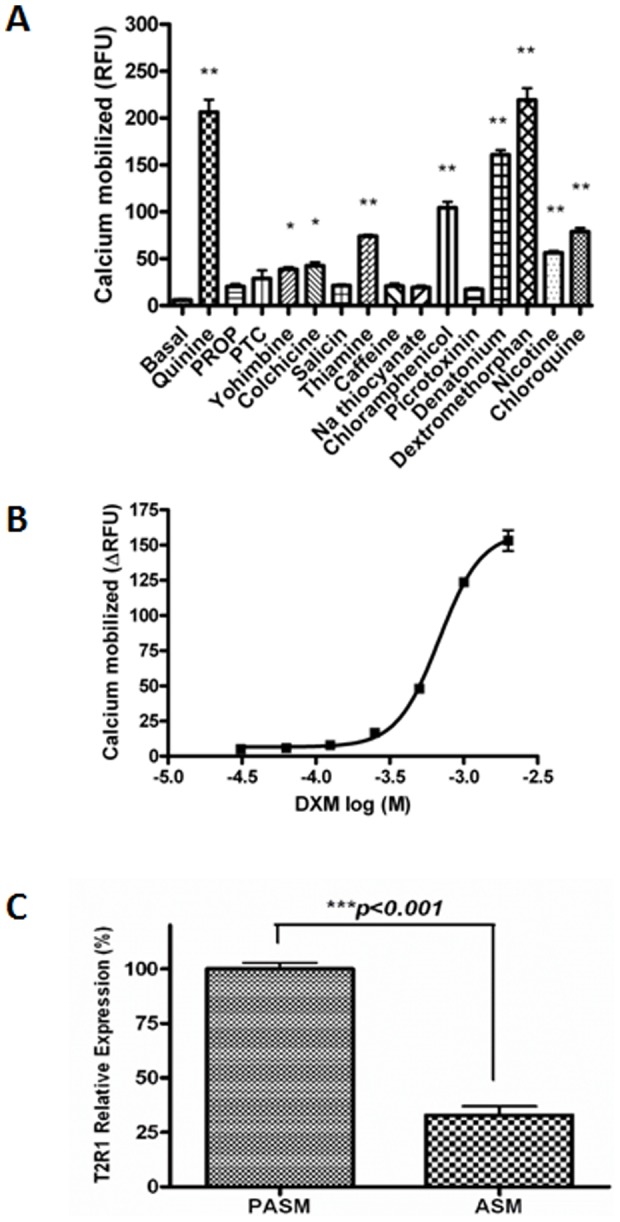
Functional response of hPASMCs to different bitter agonists. **A**. Bitter compounds of diverse structures cause increase in intracellular calcium in primary cultures of hPASMCs. Intracellular calcium [Ca^2+^]_i_ responses to 1 mM yohimbine, 2 mM quinine, DXM and nicotine, 3 mM chloramphenicol and picrotoxinin, 5 mM colchicine, thiamine, caffeine, PTC, PROP and sodium thiocyanate, and 10 mM denatonium benzoate, salicin and chloroquine. Results are means ± SEM from n = 5 done in triplicate, except for salicin and caffeine (n = 4). *p<0.05 vs control and **p<0.01 vs control. **B**. Concentration dependent changes in [Ca^2+^]_i_ of hPASMCs expressing endogenous T2R1 induced by exogenous bitter agonist DXM (log M). Data were collected from five independent experiments carried out in triplicate. For the calculation of dose response curve, signals of 10–15 wells receiving the same concentrations of same test substances were averaged, and the fluorescence changes of corresponding unstimulated cells were subtracted. An EC_50_ value of 676±90 µM for DXM in hPASMCs was calculated using Graph Pad Prism software. **C**. Relative expression levels of T2R1 in hPASMCs and hASMCs as determined by quantitative (q)-PCR. Relative expression of T2R1 in hASMCs was normalized to that in hPASMCs which was considered as 100%. Data presented are from five independent experiments done in triplicates. Results are normalized to expression of GAPDH. Values are plotted as mean ± SEM. Relative expressions were computed using 2^−ΔCT^ method. Statistical significance of T2R1 expression in hPASMCs was determined by student *t*-test, ***p<0.001 vs T2R1in hASMCs.

To determine the expression of TAS2R transcripts in hPASMCs, all 25 TAS2Rs were selected for analysis by RT-PCR. The RNA was isolated from hPASMCs, cDNA was synthesized and RT-PCR performed as described in methods. Transcripts of most of the TAS2Rs were detected in the hPASMCs, except for TAS2R16, TAS2R38, TAS2R40 and TAS2R41 (**Figure 1**). This could be due to the low copy number and/or expression of these TAS2R genes in hPASMCs. Having demonstrated that hPASMCs express TAS2R transcripts (**Figure 1**), we analyzed whether these T2Rs are functional, by stimulating the cells with several known bitter agonists [23] (**Figure 2A**). The bitter compounds used for activating these cells were yohimbine (1 mM), quinine, DXM, nicotine (2 mM each), chloramphenicol, picrotoxinin (3 mM each), colchicine, thiamine, caffeine, PTC, PROP, sodium thiocyanate (5 mM each), salicin, chloroquine and denatonium benzoate (10 mM each). A list of the T2Rs activated by these compounds is provided in supplementary section (Table S1 in File S1). The respective concentrations were selected based on their published Emax values, and keeping in view the low affinity of T2Rs for their ligands, usually in the higher micro to millimolar range [13], [23]. The increased intracellular calcium levels upon stimulation indicated that T2Rs in cultured hPASMCs are functional (**Figure 2A**). Most of these compounds were able to activate multiple T2Rs. The maximum response was seen for DXM, currently known to activate only T2R1 [23], [24]. Previously, in a site directed mutational analysis of T2R1 we have shown that DXM causes a concentration-dependent increase in intracellular calcium in cells expressing T2R1, and were able to map the DXM binding pocket on T2R1 [15]. Next, cultured hPASMCs were treated with different concentrations of DXM (Figure S1 in File S1). As shown in Figure 2B, the addition of DXM induced a concentration dependent increase in intracellular calcium in hPASMCs, with an EC_50_ value of 676±90 µM. The effect of DXM on intracellular calcium release in hASMCs was also analyzed and significantly low calcium was released with 2 mM DXM in hASMCs when compared to hPASMCs (Figure S2 in File S1).

Differences in the expression of T2R1 among hPASMCs and hASMCs were analyzed by performing qPCR in both cell types. The data demonstrated that expression of T2R1 in hPASMCs was three fold higher than that in hASMCs (**Figure 2C**). Further, qPCR analysis was done to analyze the expression pattern of T2R1 in porcine PASMCs and ASMCs (Figure S3 in File S1). Expression pattern of T2R1 in porcine PASMCs and ASMCs was similar to its expression pattern observed in human PASMCs and ASMCs, i.e. T2R1 expression in PASMCs was 3-fold higher than that observed in ASMCs.

### T2R1 knockdown in hPASMCs

**Figure 3:**
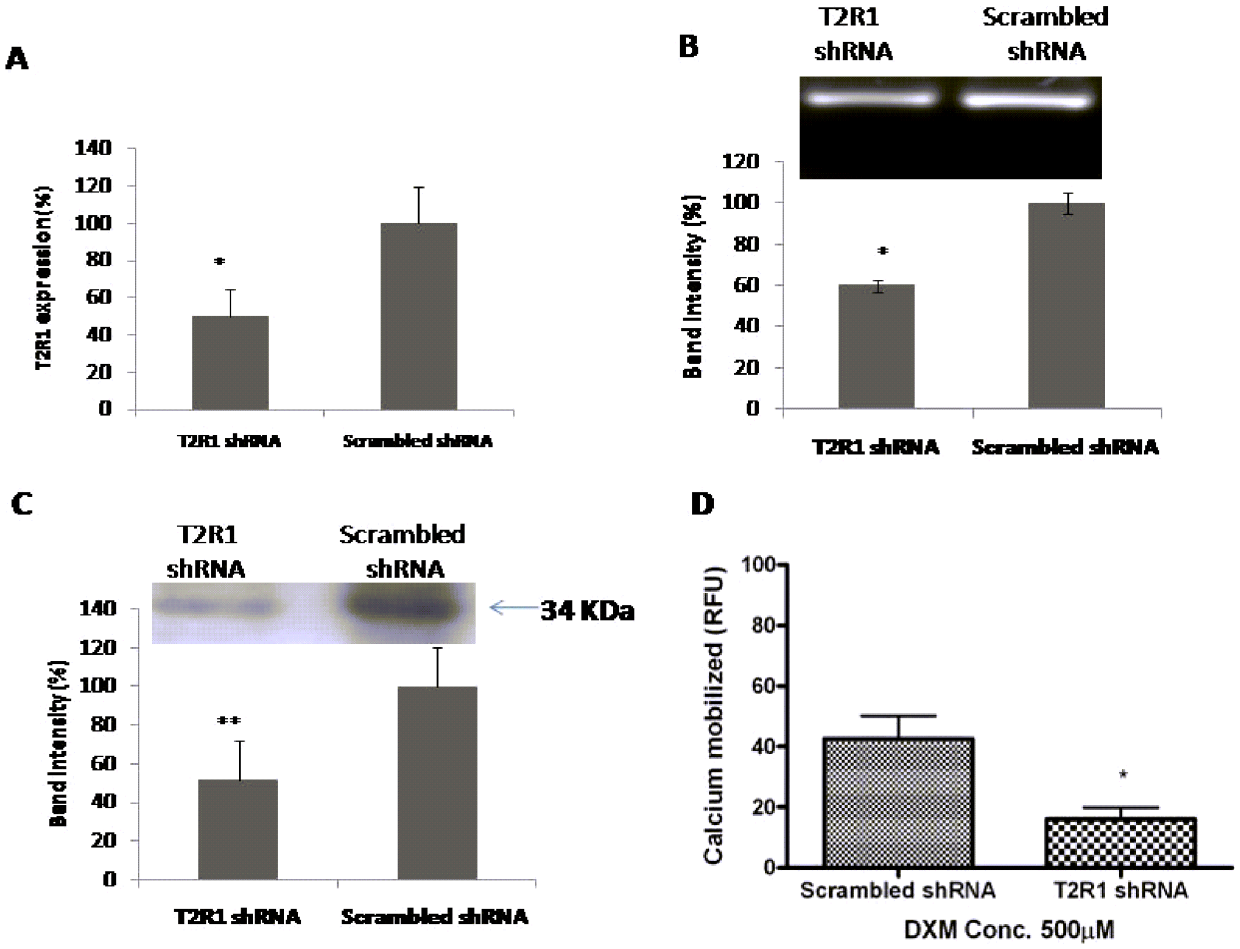
Knockdown of T2R1 in hPASMCs. **A**. Primary cultures of hPASMCs were transfected with scrambled shRNA (control) or shRNA T2R1. 48 h post-transfection, cells were used for RNA extraction and real-time PCR. Results are normalized to GAPDH expression. Percentage (%) knockdown efficiency was computed using 2^−ΔΔCT^ method. Values are mean ± SEM, n = 5. Statistical significance was determined by student *t*-test, *p<0.05 vs scrambled shRNA (control). **B**. Representative agarose gel analysis of figure 3A. Lane 1 represents T2R1-shRNA and lane 2 scrambled shRNA. Quantification of T2R1 knockdown is represented via bar graph using the densitometric analysis. Statistical significance was determined by student *t*-test, *p<0.05 vs scrambled shRNA. **C**. Western blot analysis showing T2R1 knockdown at the protein level in hPASMCs. Band intensity was normalized to expression of β-actin. Bar graph shows the quantitative analysis of receptor knockdown in the blot. Statistical significance was determined by student *t*-test, **p<0.01 vs scrambled shRNA (control). **D**. Functional effects of T2R1 knockdown in hPASMCs. hPASMCs were transfected with scrambled shRNA (control) and shRNA T2R1. 48 h post-transfection, cells were used for calcium mobilization experiment, and stimulated with 500 µM DXM. Data were collected from five independent experiments carried out in triplicate. Values are mean ± SEM, n = 5. Statistical significance was determined by student *t*-test, *p<0.05 vs scrambled shRNA (control).

**Figure 4:**
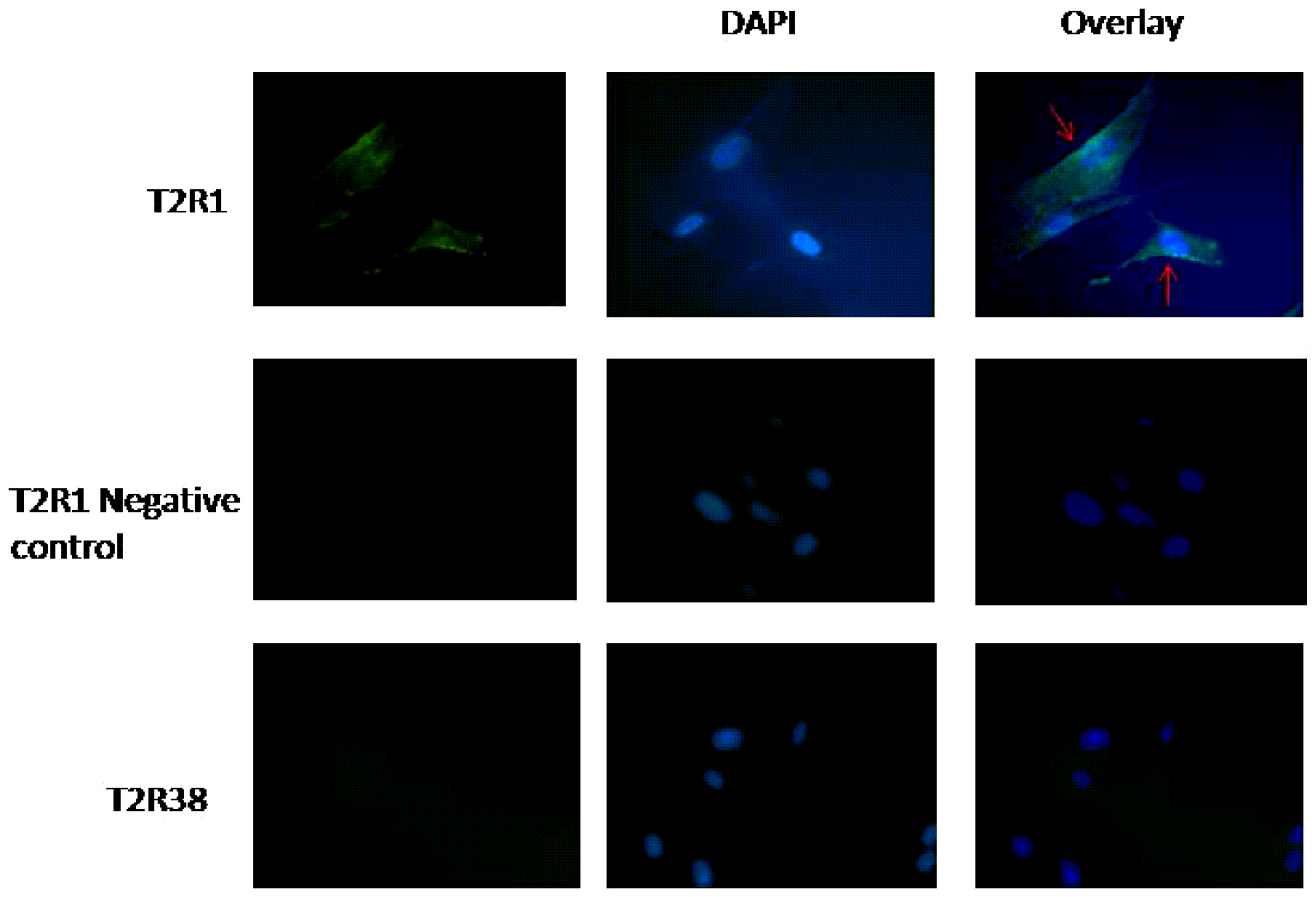
Immunofluorescence showing expression of T2R1 in hPASMCs. hPASMCs were processed by standard immunofluorescence microscopy as described in the methods. T2R1 antisera was utilized to identify the indicated protein (first row). The negative control (second row) utilized an isotype-matched non-specific IgG as the primary antibody, and T2R38 antisera (third row) showed no signals. Rabbit anti-human T2R1 was visualized with goat anti-rabbit Alexa 488 antibody (green) and nuclei were stained with DAPI (blue). Merged images show that T2R1 localized partly on the cell surface of hPASMCs cells as indicated by arrows.

To analyze if the increase in intracellular calcium level after DXM incubation was T2R1 specific, knockdown of T2R1 in hPASMCs was pursued. Transfection of hPASMCs with T2R1 specific shRNA decreased T2R1 mRNA by 50±12% compared to the scrambled shRNA control (**Figure 3A**). Agarose gel analysis of the PCR products followed by densitometric analysis revealed around ∼40% knockdown (**Figure 3B**). Immunoblot analysis revealed a decrease of ∼50% in T2R1 protein expression (**Figure 3C**). This was accompanied by an ∼50% decrease in the intracellular calcium level stimulated by the T2R1 agonist DXM (**Figure 3D**). Further, immunofluorescence microscopy of hPASMCs with polyclonal antisera directed against T2R1 revealed that the receptor was partly localized on the cell surface. However, no staining was observed for the negative controls, isotype specific IgG antisera and T2R38 (**Figure 4**).

### DXM mediated differential responses in piglet pulmonary arterial and airway rings

**Figure 5:**
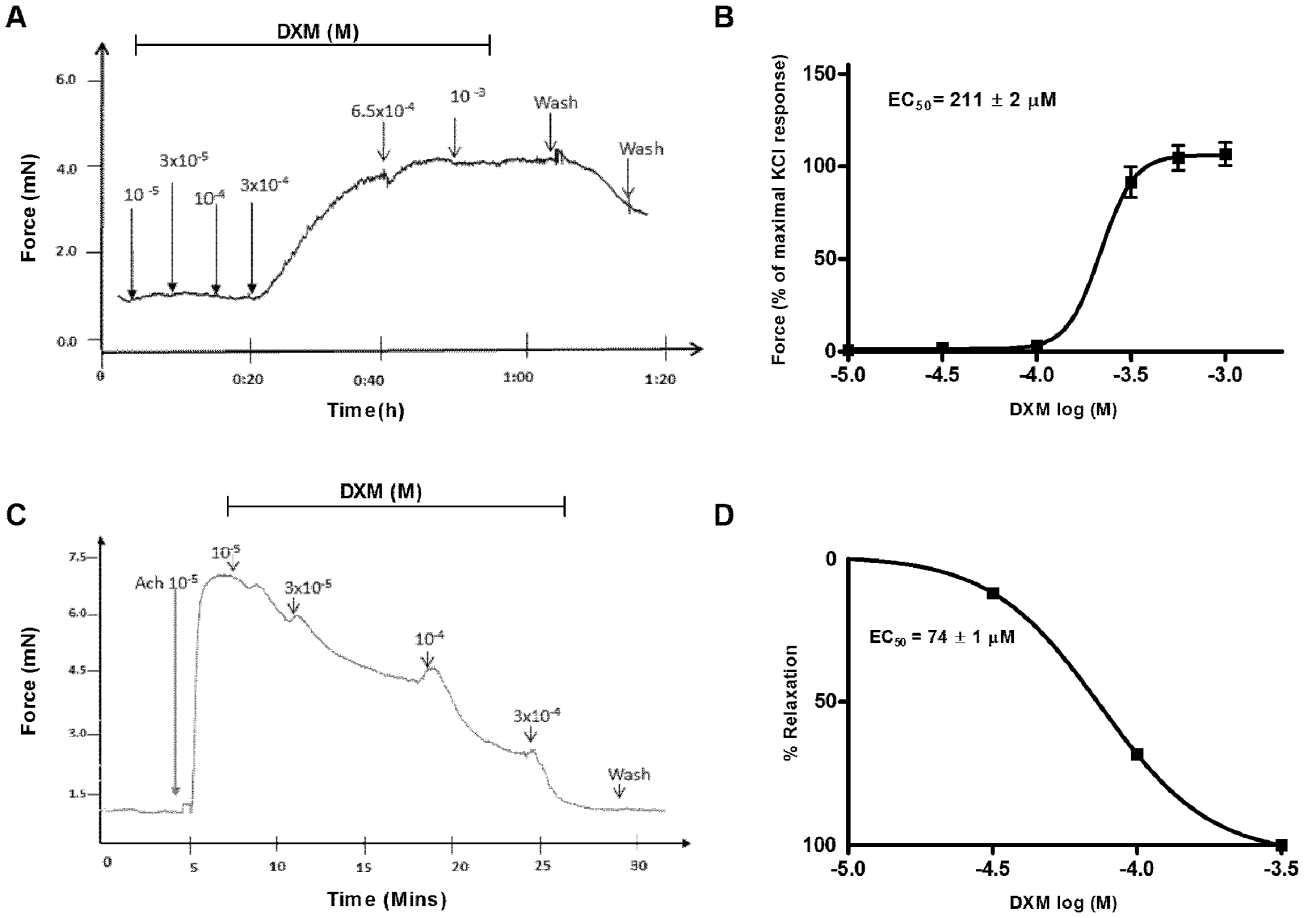
Myograph analysis of the effects of DXM on the porcine pulmonary arterial and airway rings. A. The figure represents the raw trace showing the effect of DXM (10^−5^ to 10^−3^ M) stimulation on resting tension of pulmonary artery rings. Force generation started from 300 µM, increasing slowly to plateau after 15–20 min; followed by a slight increase in force at 650 µM DXM. There was no further increase in tension even with up to 1 mM DXM. Force returned to baseline 20–30 min after 3–4 washings of pulmonary artery rings with Krebs solution. **B**. Dose response curve of DXM normalized to maximal KCl stimulation in pulmonary arterial rings. Cumulative dose response curve of DXM with highest concentration being 3×10^−4^ M and lowest 10^−5^ M on isometric tension of pulmonary artery rings. The DXM responses were normalized to maximal KCl stimulation and the EC_50_ was calculated to be 211±2 µM. The results are presented as mean ± SEM and are from a minimum of n = 15 rings from 5 piglets. **C**. Representation of raw trace of the DXM doses added to piglet airway rings precontracted with 10^−5^ M ACh. DXM completely relaxed the precontracted airways in a dose dependent manner reaching baseline at 3×10^−4^ M. **D**. Dose response curve of DXM normalized to maximal KCl stimulation in airway rings. The figure shows the effect of DXM dose response on porcine airway rings precontracted with 10^−5^ M ACh. DXM-mediated relaxation is expressed as percentage of the maximum force due to KCl stimulation and the EC_50_ calculated was found to be 74±1 µM. The results are presented as mean ± SEM and are from a minimum of n = 15 rings from 5 piglets.

Pulmonary arterial rings were optimally equilibrated at a median resting tension of 1.5 mN. To examine the DXM mediated physiological response in pulmonary arterial rings, with and without U46619 precontraction, rings were exposed to serial concentrations of DXM (10^−5^ to 10^−3^ M). Treatment of the resting arterial rings with 300 µM DXM caused noticeable force generation, which increased slowly and took 15–20 min to reach a plateau. This was followed by a slight increase in force at 650 µM of DXM, which remained stable even after adding up to 1 mM of DXM (**Figure 5A**). Next, a DXM dose response curve was constructed by normalizing the force generation to maximal KCl stimulation, and the EC_50_ was calculated to be 211±2 µM (**Figure 5B**). To confirm this vasoconstrictor response of DXM, the arterial rings were precontracted with U46619 (30 nM). DXM exposure caused contraction of precontracted rings starting from 100 µM concentration which increased till 650 µM, and then reached a plateau (Figure S4A and B in File S1). In contrast, chloroquine induced relaxation of U46619-precontracted arterial rings (30–35%). The levels of precontraction to U46619 (30 nM) in pulmonary arteries was ∼140% of KCl (50 mM)-induced contractions. We also explored the role of endothelium in this DXM-mediated functional response by using endothelium-denuded pulmonary arterial rings. DXM stimulated endothelium denuded pulmonary artery rings in a similar manner with EC_50_ of 238±1 µM (Figure S5 in File S1). In contrast to pulmonary arterial rings, DXM completely relaxed the 10 µM acetylcholine (ACh) precontracted airway rings in a dose dependent manner with an EC_50_ of 74±1 µM, and reaching baseline at 3×10^−4^ M (**Figure 5C and 5D**). Further, when ACh (10 µM) precontracted airway rings were exposed to different concentrations of chloroquine (10^−5^ to 3×10^−4^ M), it was found that chloroquine caused relaxation in a dose dependent manner (Figure S6 in File S1). Relaxant effects of DXM on airway rings and of chloroquine on airway and arterial rings are consistent with previous studies done in mouse airways, guinea pig aorta and tracheal rings and human pulmonary arteries [7], [25].

### IP_3_ measurement and analysis of MLC and phospho MLC

**Figure 6:**
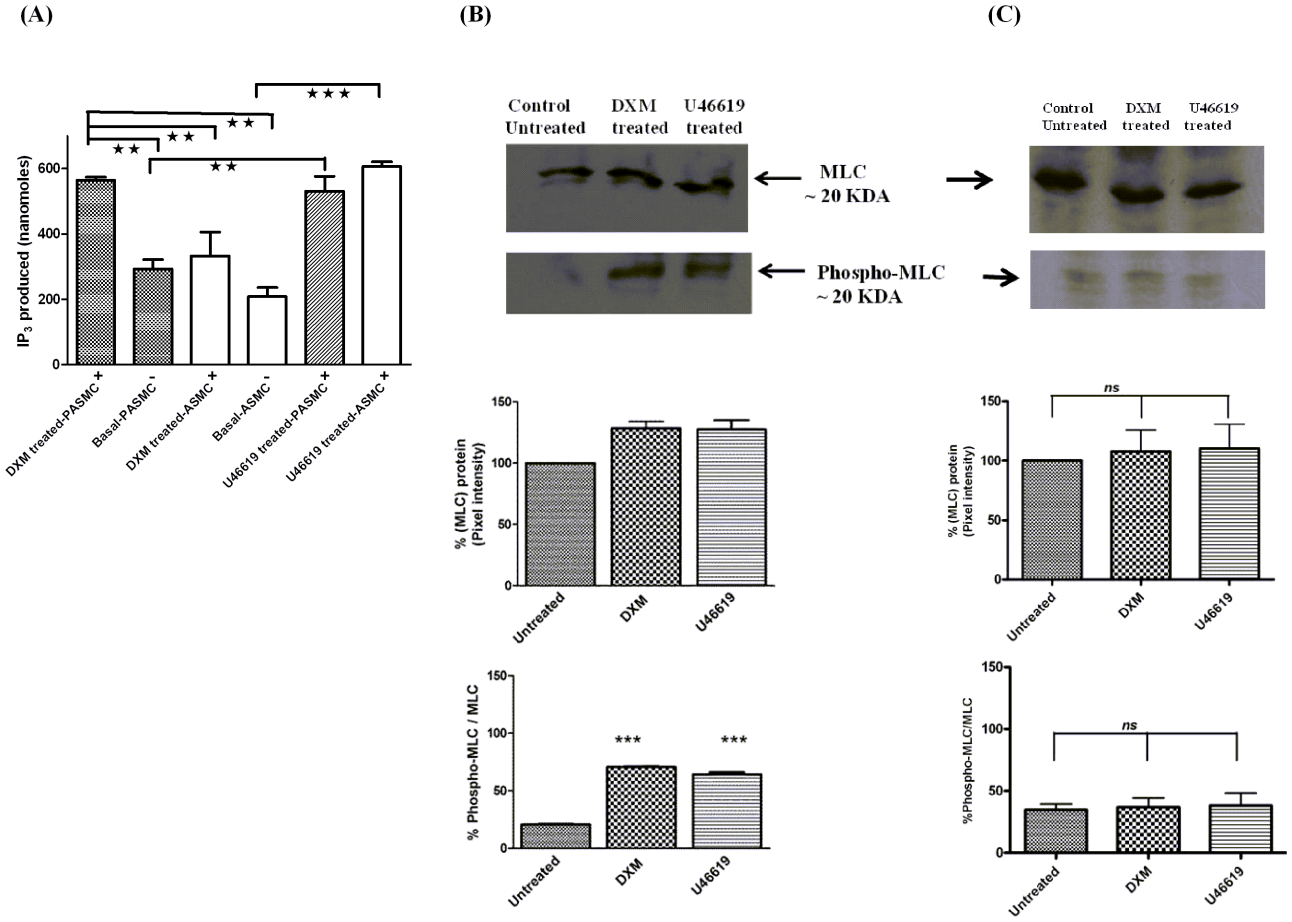
IP_3_ measurement in porcine PASMCs and ASMCs, and analysis of MLC and phospho MLC in porcine PASMCs. **A**. Bar plot representation of total IP_3_ produced (nanomoles) in pPASMCs and pASMCs upon treatment with DXM. pASMCs and pPASMCs were stimulated with DXM (500 µM) or U46619 (1 µM) which was used as a positive control. Shown are the agonist-independent or basal activity (-), and activity after stimulation (+) with agonist. Results are from a minimum of 4 independent experiments performed in triplicate. A one way ANOVA with Tukeys *post hoc* test was used to check the significance. The double asterisk indicate a significant difference in the amount of IP_3_ produced after stimulation with 500 µM DXM in pPASMCs with respect to DXM response in pASMCs at significance level of **p<0.01. Error bars represent mean ± SEM. **B**. Representative Western blot image and the respective densitometry analysis of three blots showing total MLC and phospho MLC in pPASMCs after DXM-treatment (500 µM). PASMCs treated with buffer alone or U46619 (1 µM) treated cells were used as controls. Both DXM and U46619 stimulated MLC phosphorylation. Significant results were obtained between untreated vs. DXM treated and untreated vs. U46619 treated at significance level ***p<0.001 for phospho-MLC. **C**. Representative Western blot image and the respective densitometry analysis of three blots showing total MLC and phospho MLC in pASMCs after DXM-treatment (500 µM). No significant change in total MLC expression or phospho MLC was found between untreated sample and U46619 treated or DXM treated sample. The results are from a minimum of three independent experiments (n = 3) and shown as mean + SEM.

Activation of the canonical bitter taste signaling involves: T2R-gustducin-PLCβ and IP_3_, which in turn causes an increase in global intracellular calcium levels [26]. Therefore, to investigate the contrasting mechanisms involved in DXM-mediated responses in pulmonary artery and airways, IP_3_ production was measured in human and porcine cells. In human cells, there was no effect of DXM-treatment on IP_3_ production in ASMCs as compared to untreated cells. However, significantly higher IP_3_ production was observed in DXM-treated hPASMCs, which was two-fold of DXM-treated hASMCs (Figure S7 in File S1). Similarly, significantly higher IP_3_ was obtained in DXM-treated PASMCs of porcine origin as compared to DXM-treated ASMCs (**Figure 6A**). DXM-treatment led to a small increase in IP_3_ production in porcine ASMCs, which was not significant when compared to the basal IP_3_ levels.

One of the key intracellular signaling mechanisms underlying vascular smooth muscle cell contraction is the phosphorylation status of the 20 kDa MLC [27], [28]. Contraction is induced by the increased phosphorylation of MLC. Therefore, we measured the phosphorylation status of MLC in DXM treated and untreated porcine PASMCs and ASMCs. U46619, a potent vasoconstrictor, was used as a positive control. DXM treatment of PASMCs led to increase in phosphorylation of MLC compared to the untreated PASMCs (**Figure 6B**). However, DXM-treatment of porcine ASMCs did not cause any significant change in the MLC phosphorylation status (**Figure 6C**).

### Effect of DXM on superoxide production

In vivo studies in rat models, and in vitro studies using human aortic endothelial cells [29] and neuron-glial cell cultures [30] showed that DXM acts as a NADPH oxidase inhibitor causing a decrease in superoxide production. Studies in rats show that superoxide production by NADPH oxidase has a role in the development of hypertension and generation of vasoconstrictor responses in aorta [31]. To investigate the inhibitory role of DXM on NADPH oxidase activity in hPASMCs, superoxide production in hPASMCs was measured. DXM (0.5 mM) treatment of hPASMCs did not cause any significant modification in superoxide production compared to the untreated group (Figure S8 in File S1).

## Discussion

Stimulation of T2Rs in the lumen of gastrointestinal tract may condition the future avoidance of similar ingesta through the process of conditioned flavor avoidance, thus providing a second line of defense against potentially toxic compounds [32], [33]. Previous studies have found expression of T2Rs in ASM, where they caused marked relaxation in intact airways and might represent a new target for bronchodilation in asthma and obstructive airway disease [7], [25]. A recent report also identified T2Rs on motile cilia of airway epithelial cells and in the anterior nasal cavity, where they increase the ciliary beat frequency [6], promote sneezing, and regulate respiratory rate against noxious inhalants [5]. While this manuscript was under revision, the expression of four T2Rs was demonstrated in human pulmonary arteries [25]. However, the presence and functional significance of all T2Rs in the pulmonary vasculature remains unreported.

In this study, a majority of the TAS2R transcripts were detected in hPASMCs. Functional screening revealed intracellular calcium responses to stimulation with a number of bitter agonists (**Figure 2A**). Among the 15 agonists tested, hPASMCs showed the maximum calcium mobilization response to DXM treatment, followed by quinine. Interestingly, our previous taste sensory analysis showed that quinine and DXM are the most intense bitter tasting compounds [15], [34]. There were couple of reasons for choosing DXM in the current study; compared to quinine, which activates nine T2Rs, DXM was shown to activate only T2R1 [23], [24]. In addition, recent and extensive structure-function studies pursued on T2R1 led to the pharmacological characterization of the potency of DXM, and identification of the ligand binding pocket on T2R1 [15], [34]. Using different techniques including qPCR, receptor knockdown using shRNA, Western blot analysis of the decrease in T2R1 expression in knockdown cells, and immunofluorescence, we confirmed the expression of T2R1 in hPASMCs, and link expression to DXM mediated intracellular calcium signaling.

Despite the current lack of knowledge on endogenous ligands for T2Rs, the effect of the tested agonist(s) in the porcine arterial tissues provides relatively clear insight into which receptors are activated. DXM displays a previously unknown pharmacological activity in the pulmonary circuit, causing T2R activation leading to vasoconstrictor responses. Due to feasibility concerns in obtaining intact human lung tissues and similar expression pattern of T2R1 in human and porcine PASMCs and ASMCs, we tested the *ex vivo* effect of DXM on porcine pulmonary arterial and airway rings. We found that DXM treatment of the pulmonary arterial rings caused vasoconstriction with an EC_50_ of 211±2 µM (**Figure 5B**). The effect of DXM on U46619-precontracted arterial rings was also analyzed in this study. DXM, starting from 100 µM, caused contraction of precontracted rings (Figure S4 in File S1). This might be a synergistic effect of DXM and U46619 on the pulmonary artery. The role of endothelium was also analyzed in this DXM-mediated response of pulmonary arterial rings. DXM-treatment of endothelium-denuded pulmonary arterial rings did not show much change in EC_50_ value of DXM (238±1 µM, Figure S5 in File S1). Study by Manson *et al*. showed that bitter agonists, chloroquine, DXM, and noscapine lead to relaxation of precontracted human pulmonary arteries [25]. However, minimal relaxation was observed with DXM-treatment in their study following U46619-induced pre-contraction. Our results contradict this recently published data on DXM in the human pulmonary artery [25]. A possible explanation for the different results may be that Manson *et al*. did not use DXM concentration beyond 100 µM. Furthermore, the role of T2R1 in mediating DXM responses was not considered in that study. In contrast, chloroquine led to relaxation of precontracted arterial rings (Figure S4 in File S1). This might indicate that DXM has a different mechanism of action in vascular smooth muscle than chloroquine. Interestingly, DXM caused relaxation of the airway rings with EC_50_ value of 74±1 µM. This later effect of DXM in the airways, acting through T2Rs, was recently demonstrated [25]. T2Rs may be developmentally regulated and/or differentially expressed among species and age groups [35]. We did not analyze the relative expression of all T2Rs in porcine, but our results demonstrate similar expression pattern of T2R1 in human and porcine cells. Increased expression of T2R1 in porcine PASMCs correlates with the increased IP_3_ production upon DXM treatment, which caused increase in MLC phosphorylation and contraction observed in arterial rings (**Figure 5** **and** **6**). Whereas, DXM treatment did not produce any significant change in IP_3_ generation in porcine ASMCs which correlates with the low T2R1 expression and the observed effect of relaxation in airway rings. Hence, we speculate that this receptor might be predominantly involved in DXM-mediated contraction of pulmonary arterial rings. Interestingly, our myographic data suggests that the dose of DXM plays a role in the observed effects; at low concentrations (74 µM), DXM leads to relaxation of airways, whereas, higher concentrations (211 µM) cause contraction of the pulmonary artery. This might be a protective reflex for regulating the vascular tone when excess noxious irritants are ingested/inhaled by the body. These results suggest that DXM mediates differential effects in different body tissues.

We examined pulmonary arterial reactivity in vessels and airways from newborn piglets. While reactivity of the neonatal pulmonary vasculature can differ from that of the adult pulmonary circuit, the neonatal pulmonary vasculature is in general more sensitive to vasospastic agents than in the adult; this vasospasm can precipitate severe pulmonary hypertension (persistent pulmonary hypertension of the newborn) in response to constrictors including hypoxia, inflammation or noxious stimuli such as bitter tastants. Porcine pulmonary vessels were used as we have characterized the pulmonary hypertensive model in this species [36], [37]. The neonatal system is unique, as the pulmonary circuit is dilating, naturally. As such, if a given drug has a vasoconstrictor effect, neonates offer a unique opportunity to test it. In this study, the observed effects of DXM on the neonatal porcine arterial rings suggest it acts as a vasoconstrictor. The presence of T2Rs in the neonatal pulmonary circuit in particular may be developmentally significant, in view of the constriction of the pulmonary circuit in utero, and its abrupt need for dilation post-birth; endogenous T2R ligands may pose a significant threat to normal neonatal pulmonary vasodilation.

**Figure 7:**
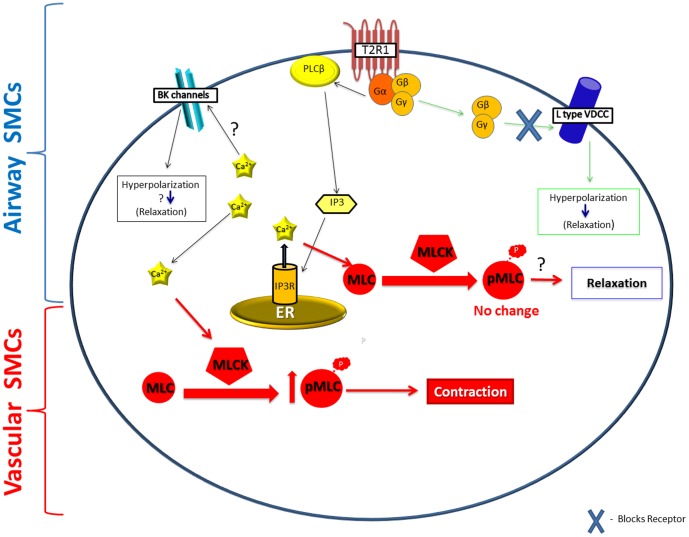
Schematic representation of the contrasting effects of DXM-induced vasoconstriction in PASMCs and relaxation in ASMCs. In our model we propose that in PASMCs DXM activates the canonical T2R signaling cascade to cause significant increase in IP_3_ production resulting in increase in global [Ca^2+^]_i_ levels. The increased [Ca^2+^]_i_ subsequently leads to activation of myosin light chain kinase (MLCK) thus resulting in an increase in the phosphorylated form of MLC (pMLC). Increase in pMLC ultimately leads to the constrictor effects observed in the pulmonary arterial rings. The molecular mechanism(s) underlying T2R mediated relaxation of the airways was studied by different groups. Deshpande DA, *et al*, [7] showed that T2R activation results in localized [Ca^2+^]_i_ mobilization which opens up large-conductance Ca^2+^ activated K+ (BK_ca_) channels leading to ASM membrane hyperpolarization and relaxation. Recently, Zhang C, *et al*, [26] demonstrated that activation of T2Rs in airways (resting state) leads to increase in global [Ca^2+^]_i_ levels, however, these are not sufficient to impact airway contractility. In a second pathway, they proposed bitter tastants inhibit L-type voltage-dependent Ca^2+^ channels (VDCCs) via a G-protein βγ dependent process, to induce bronchodilation of pre-contracted ASM.

To investigate the molecular mechanisms underlying the contrasting effects: DXM-induced contraction of pulmonary artery and relaxation of airway, IP_3_ production was measured in both human and porcine PASMCs and ASMCs after treatment with DXM. There was no effect of DXM on IP_3_ production in hASMCs, whereas, significantly increased IP_3_ production was observed in hPASMCs (Figure S7 in File S1), which confirms that higher calcium is generated to cause a contractile response in PASMCs. Similar results were obtained in porcine cells where significantly increased IP_3_ was observed after DXM-treatment of PASMCs when compared to treated ASMCs (**Figure 6A**). These contrasting effects of DXM might explain the observed difference in EC_50_ values of DXM in contracting pulmonary artery and relaxing airways. Since phosphorylation of MLC is one of the key intracellular signaling mechanisms underlying vascular smooth muscle cell contraction, we also examined the expression of MLC and phospho MLC with and without DXM treatment in porcine PASMCs. DXM treatment of PASMCs led to an increase in MLC phosphorylation (**Figure 6B**). We, thus, propose that in DXM treated PASMCs, significant IP_3_ is generated to cause phosphorylation of MLC, leading to a contractile response (**Figure 7**). The relative expression levels of T2R1 in human and porcine PASMCs correlate with the increased IP_3_ production after DXM treatment (Table S2 in File S1).

In conclusion, our results demonstrate that DXM causes vasoconstriction in the pulmonary arterial system by activating endogenous T2Rs. Bitter stimuli can enter the pulmonary circuit after ingestion, during inhalation, or generated during pathological conditions. The vasoconstrictor response mediated by T2R1 activation can be an additional defensive mechanism of the body, to detect and eliminate noxious and harmful stimuli. The novel role of T2Rs in vasoconstriction reported here adds to the growing body of evidence, which suggest T2Rs expressed in extra-oral tissues as mediators of off-target effects of diverse bitter tasting pharmaceuticals.

## Supporting Information

File S1 **Table S1, A list of bitter taste receptors activated by the compounds used in the study. Table S2, DXM mediated effects on T2Rs expressed in PASMCs, ASMCs, pulmonary artery and airway rings. Figure S1, Representative calcium traces for primary cultures of hPASMCs stimulated with different concentrations of DXM or assay buffer (bottom trace).** The calcium mobilized (Relative Fluorescence Units or RFUs) was detected using the calcium sensitive dye Fluo-4 NW (Invitrogen), and fluorescence measured using the automated Flex Station 3 microplate reader as described before [15], [34], [38]. In brief, the basal calcium mobilized was measured for the first 20 sec in all the 8 wells (one column) of a 96 well plate, followed by the simultaneous addition of different concentrations of the test compound, shown by arrows in the figure, to all the 8 wells by the in-built automated dispenser in Flex Station 3. Then calcium traces were recorded for the next 180 sec. **Figure S2, Comparison of intracellular calcium release in hPASMCs and hASMCs in response to different concentrations of DXM.** **A**. Concentration-dependent changes in [Ca^2+^]_i_ of hPASMCs and hASMCs induced by different concentrations of bitter agonist DXM (log M). Data were collected from 3–5 independent experiments carried out in triplicate. Dose response curves were generated using Graph Pad Prism software. **B**. Bar graph showing difference in intracellular calcium release in hPASMCs and hASMCs in response to 2 mM DXM (E_max_ concentration). Significant calcium release was observed in hPASMCs in comparison to hASMCs with significance level of *p<0.05. **Figure S3, Quantification of T2R1 expression in human and porcine cells. A. Relative expression level of T2R1 in porcine PASMCs and ASMCs as determined by quantitative (q)-PCR.** T2R1 expression in porcine PASMCs was considered as 100% and relative expression of T2R1 in ASMCs was normalized to it. **B**. **Relative expression of T2R1 in human and porcine PASMCs.** The relative expression of T2R1 in porcine PASMCs was normalized to that of hPASMCs, which was considered as 100%. Data presented are from five independent experiments done in triplicates. Results are normalized to the expression of GAPDH. Values are plotted as mean ± SEM. Relative expressions were computed using 2^−ΔCT^ method. Student's *t*-test was used to check the significance. **Figure S4, Myograph analysis of the effects of DXM on U46619 precontracted porcine pulmonary arterial rings.** **A**. Raw traces showing effect of DXM (10^−5^ to 6.5×10^−5^ M) stimulation on resting tension of U46619 (30 nM) precontracted pulmonary artery rings. Force generation started from 100 µM and reaching a plateau after 650 µM DXM. **B**. Effects of DXM and chloroquine on precontracted porcine arterial rings. Data are representative of n = 6 rings and presented as means ± S.E.M. The levels of precontraction to U46619 (30 nM) in pulmonary arteries was ∼140% of KCl (50 mM)-induced contractions. **Figure S5, Myograph analysis of the effects of DXM on endothelium-denuded porcine pulmonary arterial rings.** Dose response curve of DXM normalized to maximal KCl stimulation in pulmonary arterial rings. The figure represents a cumulative dose response curve of DXM with highest concentration being 10^−3^ M and lowest 10^−5^ M on isometric tension of pulmonary artery rings. The DXM responses were normalized to maximal KCl stimulation and the EC_50_ was calculated to be 238±1 µM. The results are presented as mean ± SEM and are from a minimum of n = 15 rings from 5 piglets. **Figure S6, Effects of bitter compound chloroquine on porcine airway rings.** Piglet airway rings were contracted with 10 µM of acetylcholine (ACh) and then chloroquine (10 µM–300 µM) was added to the bath as shown, resulting in relaxation in a dose dependent manner. Results are representative of five independent experiments and are from a minimum of n = 15 rings from 5 piglets. **Figure S7, IP_3_ produced in human PASMCs and ASMCs.** Bar plot representation of total IP_3_ produced (nanomoles) in hPASMCs and hASMCs upon treatment with DXM. hASMCs and hPASMCs were stimulated with T2R1 agonist DXM (500 µM) and the vasoconstrictor U46619 (TP agonist, 1 µM),which was used as a positive control. Shown are the agonist-independent or basal activity (-), and activity after stimulation (+) with agonist. Results are from a minimum of four independent experiments performed in triplicates. A one way ANOVA with Tukey's *post hoc* test was used to check the significance level of the amount of IP_3_ produced. DXM treatment caused no change in IP_3_ level in hASMCs, whereas, a significantly increased IP_3_ was observed in DXM-treated hPASMCs, as compared to hASMCs, at significance level of ***p<0.001. Error bars represent mean ± SEM. **Figure S8, Effect of DXM on superoxide production in hPASMCs.** hPASMCs were treated with 0.5 mM DXM or media alone for 4 hours. DHE fluorescence assay was done to measure the superoxide production as described in methods. Superoxide production was expressed as fluorescence intensity and as the mean ± SEM of three independent experiments performed in triplicates.
(DOCX)
