# Supplementary material for: Dextromethorphan Mediated Bitter Taste Receptor Activation in the Pulmonary Circuit Causes Vasoconstriction
Source: PLoS One. 2014 Oct 23;9(10):e110373. doi: 10.1371/journal.pone.0110373 (PMC4207743; doi:10.1371/journal.pone.0110373)
Supplement: File S1 — Table S1, A list of bitter taste receptors activated by the compounds used in the study. Table S2, DXM mediated effects on T2Rs expressed in PASMCs, ASMCs, pulmonary artery and airway rings. Figure S1, Representative calcium traces for primary cultures of hPASMCs stimulated with different concentrations of DXM or assay buffer (bottom trace). The calcium mobilized (Relative Fluorescence Units or RFUs) was detected using the calcium sensitive dye Fluo-4 NW (Invitrogen), and fluorescence measured using the automated Flex Station 3 microplate reader as described before [15], [34], [38]. In brief, the basal calcium mobilized was measured for the first 20 sec in all the 8 wells (one column) of a 96 well plate, followed by the simultaneous addition of different concentrations of the test compound, shown by arrows in the figure, to all the 8 wells by the in-built automated dispenser in Flex Station 3. Then calcium traces were recorded for the next 180 sec. Figure S2, Comparison of intracellular calcium release in hPASMCs and hASMCs in response to different concentrations of DXM. A. Concentration-dependent changes in [Ca2+]i of hPASMCs and hASMCs induced by different concentrations of bitter agonist DXM (log M). Data were collected from 3–5 independent experiments carried out in triplicate. Dose response curves were generated using Graph Pad Prism software. B. Bar graph showing difference in intracellular calcium release in hPASMCs and hASMCs in response to 2 mM DXM (Emax concentration). Significant calcium release was observed in hPASMCs in comparison to hASMCs with significance level of *p<0.05. Figure S3, Quantification of T2R1 expression in human and porcine cells. A. Relative expression level of T2R1 in porcine PASMCs and ASMCs as determined by quantitative (q)-PCR. T2R1 expression in porcine PASMCs was considered as 100% and relative expression of T2R1 in ASMCs was normalized to it. B. Relative expression of T2R1 in human and porcine PASMCs. The relative expression of [file pone.0110373.s001.docx]

**Supplementary Information**

**Table S1. A list of bitter taste receptors activated by the compounds used in the study [1,2].**

| **Bitter Compound** | **Bitter taste receptor (T2R)** |
| --- | --- |
| Quinine | T2R4, T2R7, T2R10, T2R14, T2R39, T2R40, T2R43, T2R44, T2R46 |
| PROP, PTC | T2R38 |
| Yohimbine | T2R1, T2R4, T2R10, T2R38, T2R46 |
| Colchicine | T2R4, T2R39, T2R46 |
| Salicin | T2R16 |
| Thiamine | T2R1, T2R39 |
| Caffeine | T2R7, T2R10, T2R14, T2R43, T2R46 |
| Sodium thiocyanate | T2R1, T2R38 |
| Chloramphenicol | T2R1, T2R8, T2R10, T2R39, T2R43, T2R46 |
| Picrotoxinin | T2R1, T2R10, T2R14, T2R46, T2R47 |
| Denatonium benzoate | T2R4, T2R8, T2R10, T2R13, T2R39, T2R43, T2R46, T2R47 |
| Dextromethorphan | T2R1 |
| Chloroquine | T2R3, T2R10, T2R39 |

**Table S2. DXM mediated effects on T2Rs expressed in PASMCs, ASMCs, pulmonary artery and airway rings.**

| **Parameter** | **PASMCs** | | **ASMCs** | |
| --- | --- | --- | --- | --- |
|  | **Neonatal pig** | **Adult human** | **Neonatal pig** | **Adult human** |
| **T2R1 mRNA level ^¶^**  (Relative expression %) | 60 | 100 | 20 | 30 |
| **IP_3_ produced (nmoles)**  (DXM treated)  (Basal or untreated) | 565 ± 9  294 ± 28 | 709 ± 31  320 ± 40 | 333 ± 50  210 ± 27 | 336 ± 18  318 ± 17 |
| ***Ex vivo* effect**  (Myographic studies in arterial or airway rings) | Contraction (Pulmonary arterial rings) | Not known | Relaxation (Airway rings) | Relaxation^*^ (Airway rings) |

**^¶^** This is the only T2R characterized by structure-function studies to interact with DXM.

**^*^** Shown in previous studies [3].

**References**

1. Meyerhof W, Batram C, Kuhn C, Brockhoff A, Chudoba E, et al. (2010) The molecular receptive ranges of human TAS2R bitter taste receptors. Chem Senses 35: 157-170.

2. Born S, Levit A, Niv MY, Meyerhof W, Behrens M (2013) The human bitter taste receptor TAS2R10 is tailored to accommodate numerous diverse ligands. J Neurosci 33: 201-213.

3. Pulkkinen V, Manson ML, Safholm J, Adner M, Dahlen SE (2012) The bitter taste receptor (TAS2R) agonists denatonium and chloroquine display distinct patterns of relaxation of the guinea pig trachea. Am J Physiol Lung Cell Mol Physiol 303: L956-966.


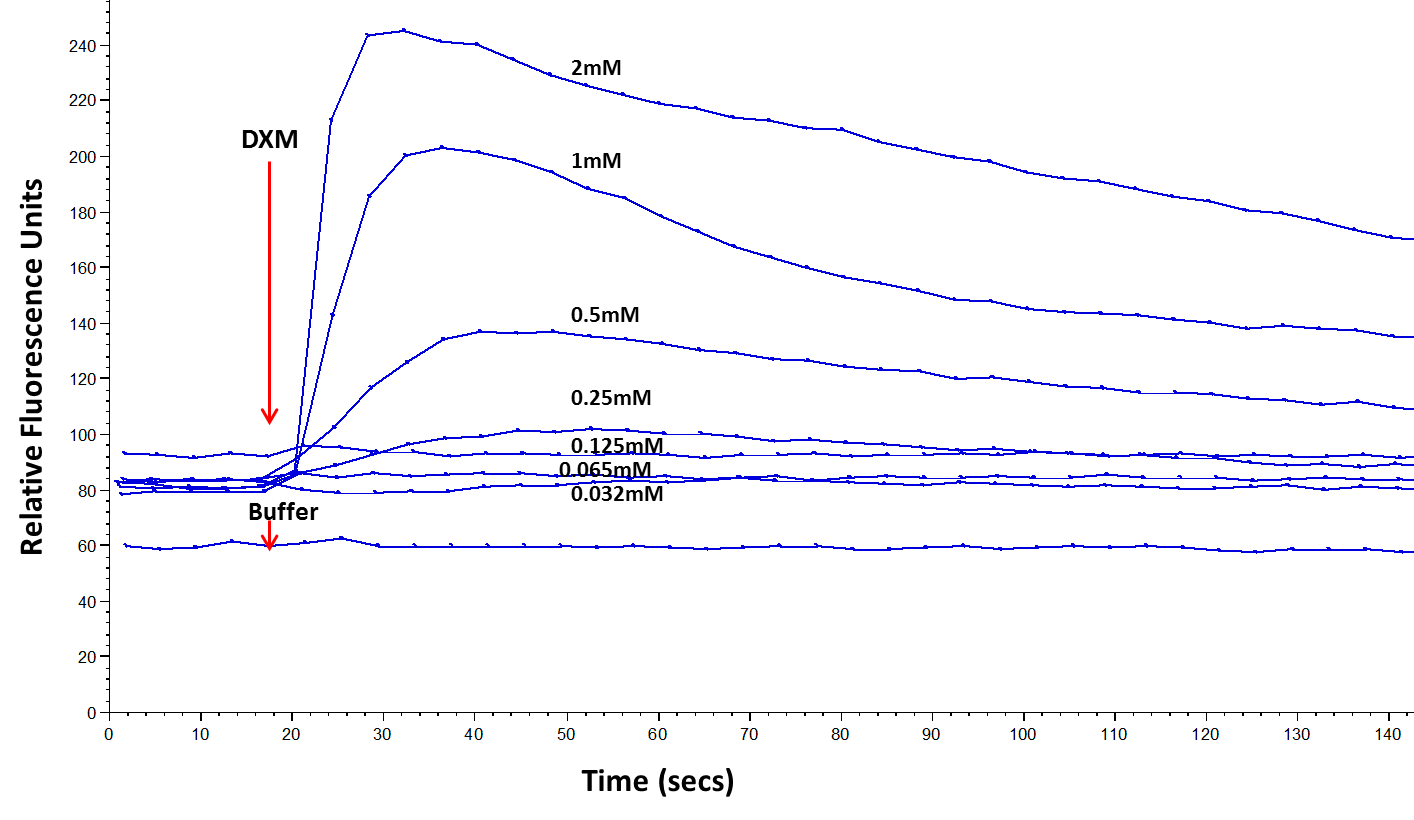


**Figure S1**

**
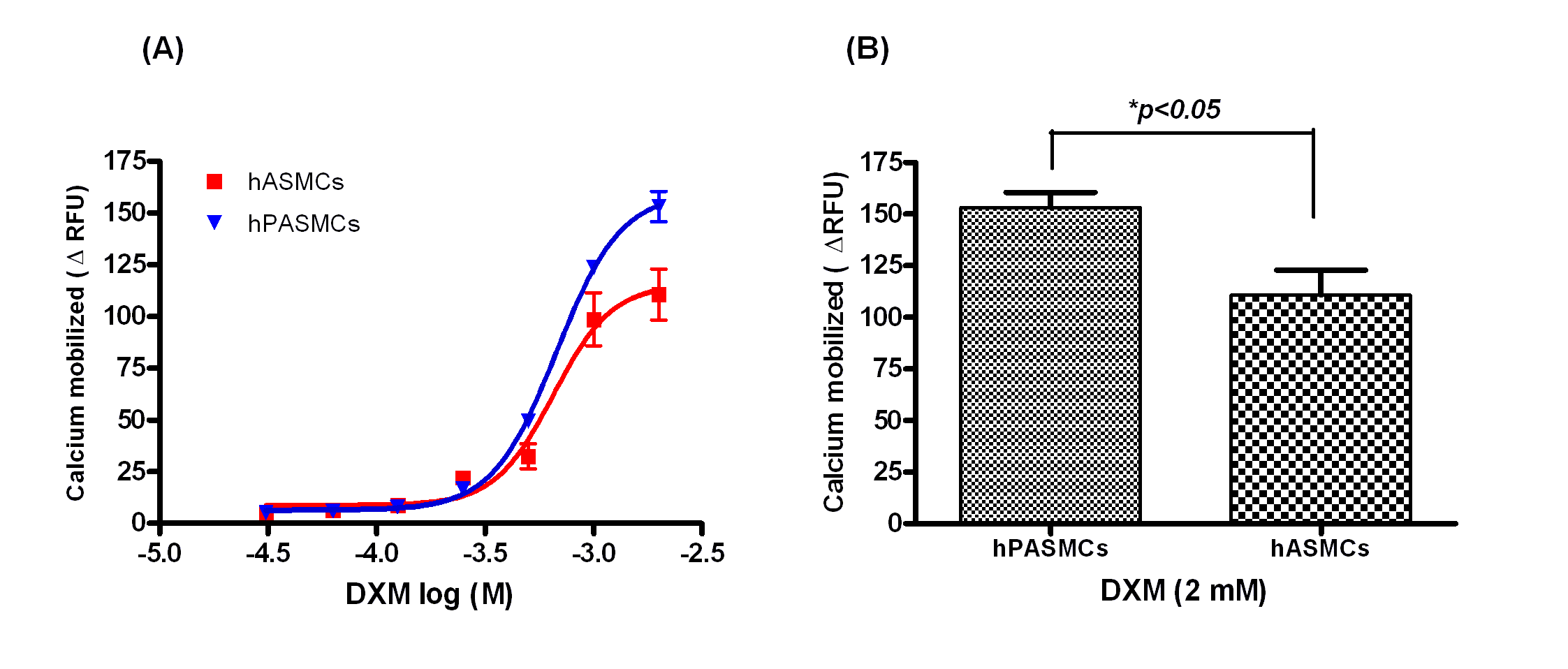
**

**Figure S2**

**
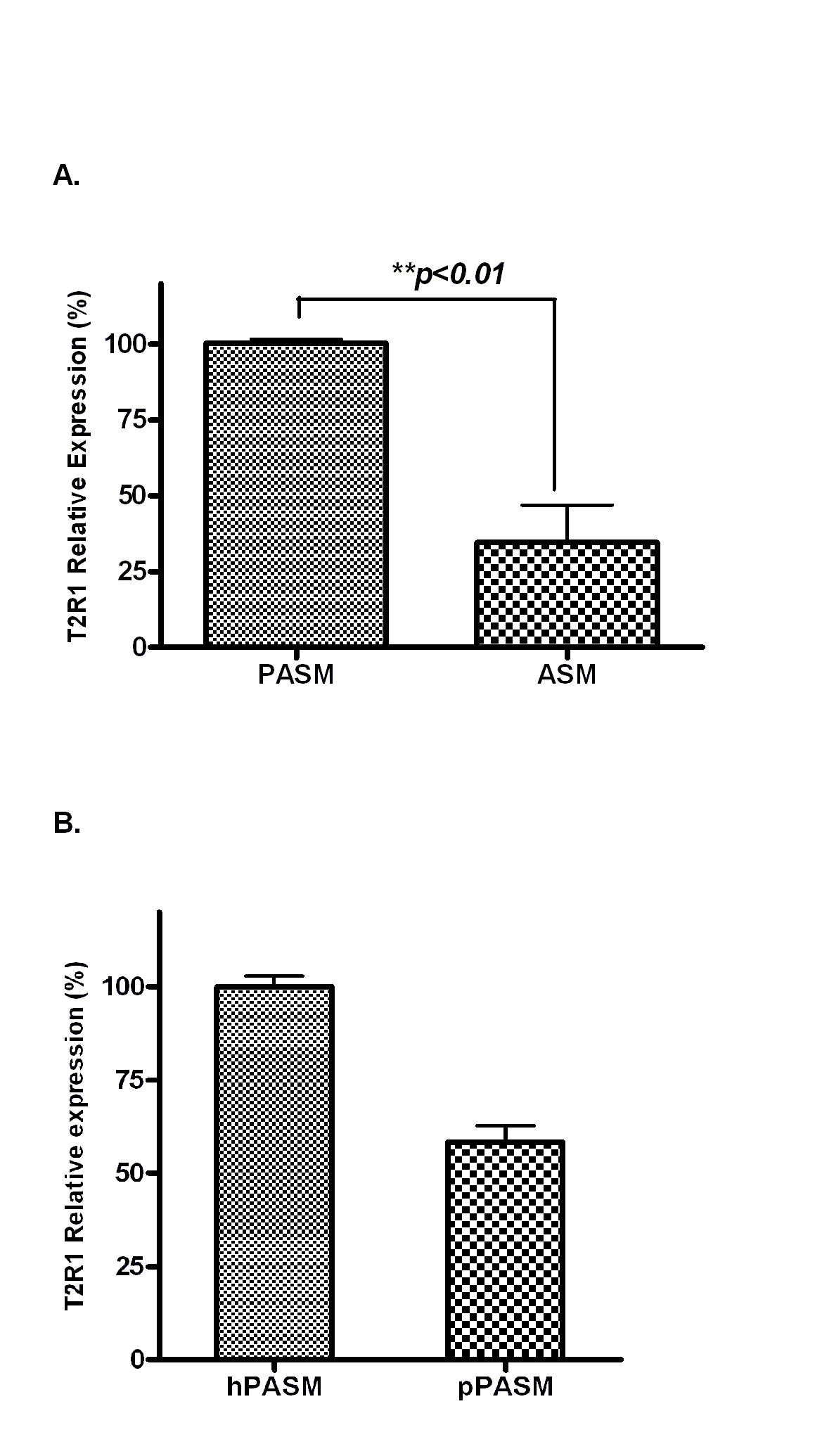
**

**Figure S3**

**
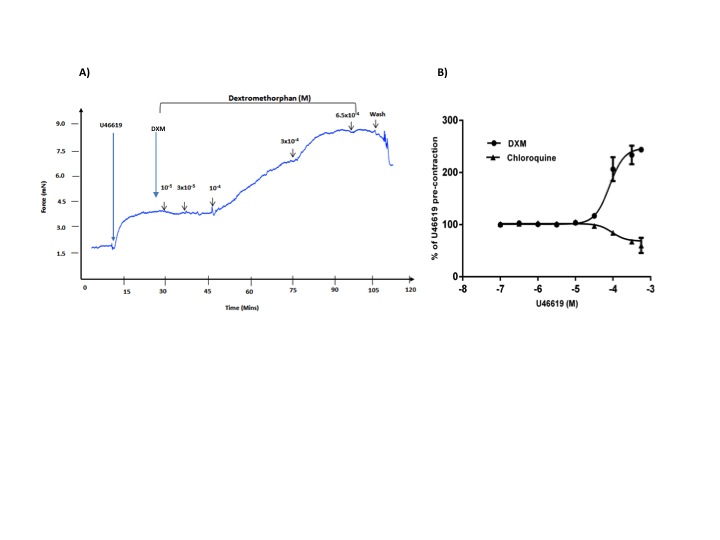
**

**Figure S4**

**
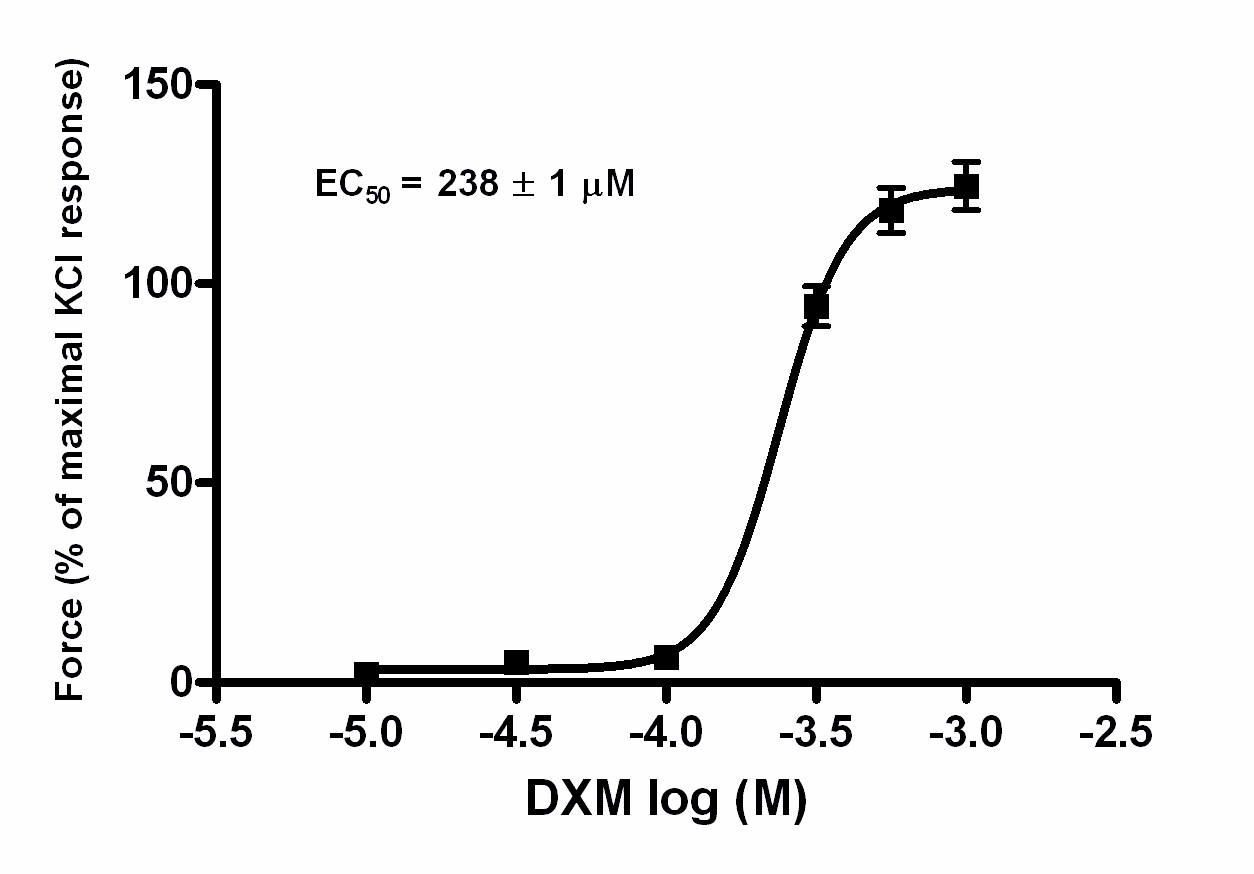
**

**Figure S5**

**
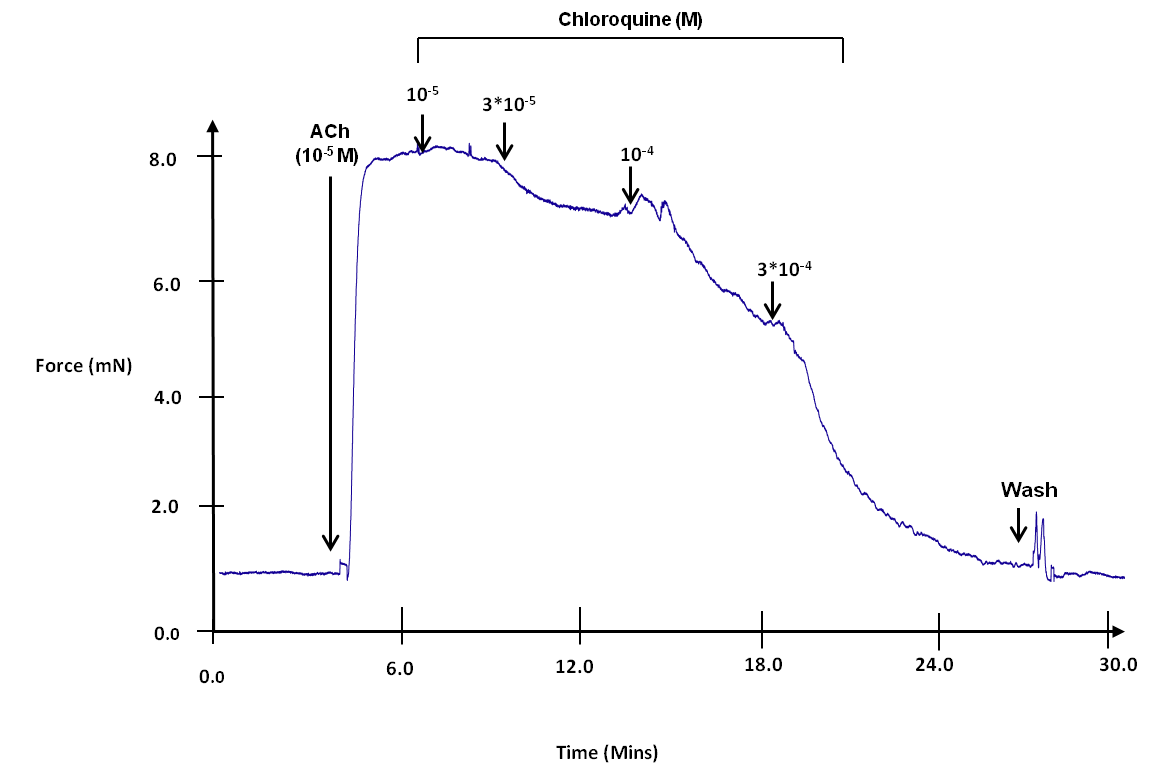
**

**Figure S6**

**
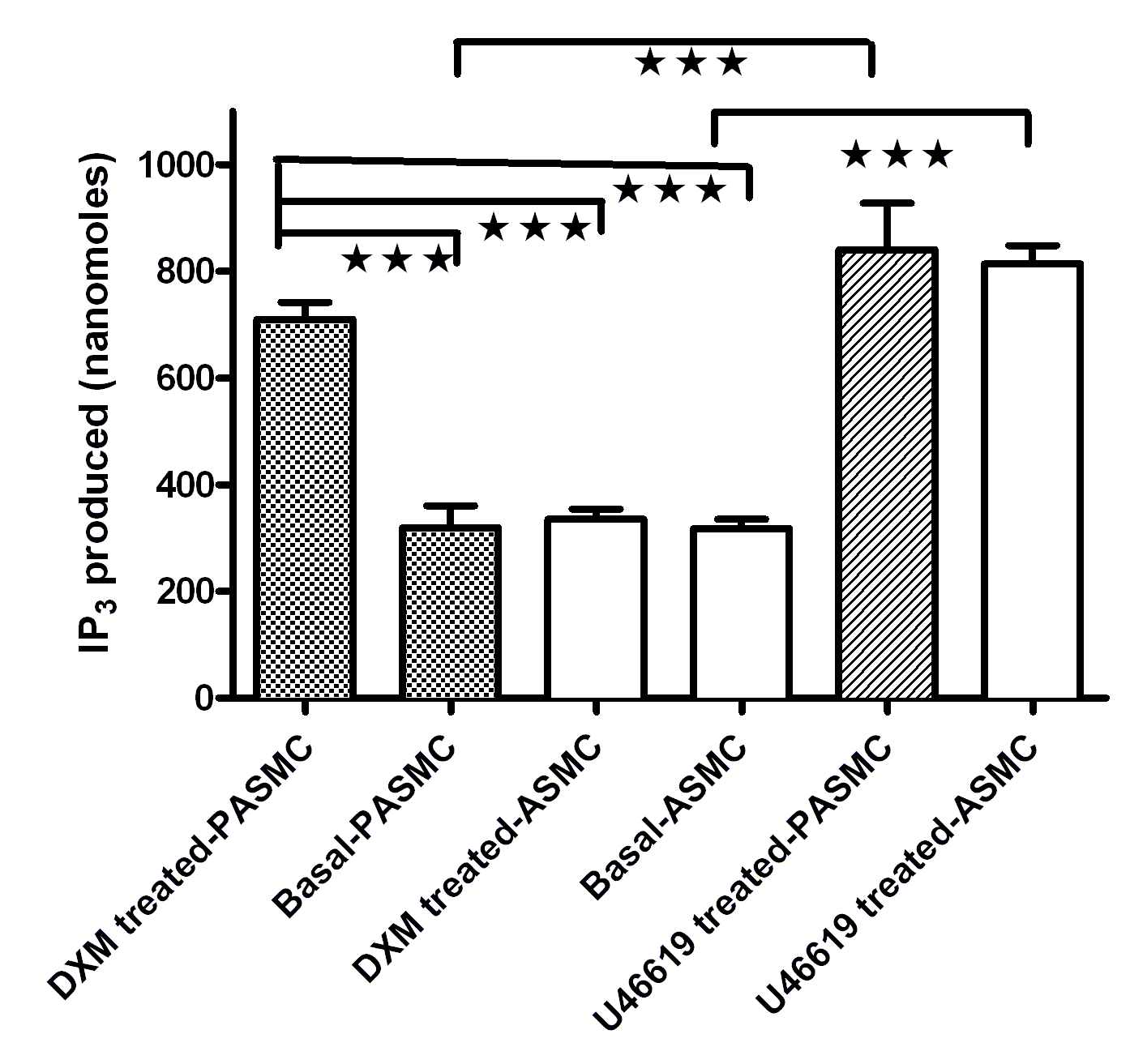
**

**Figure S7**

**
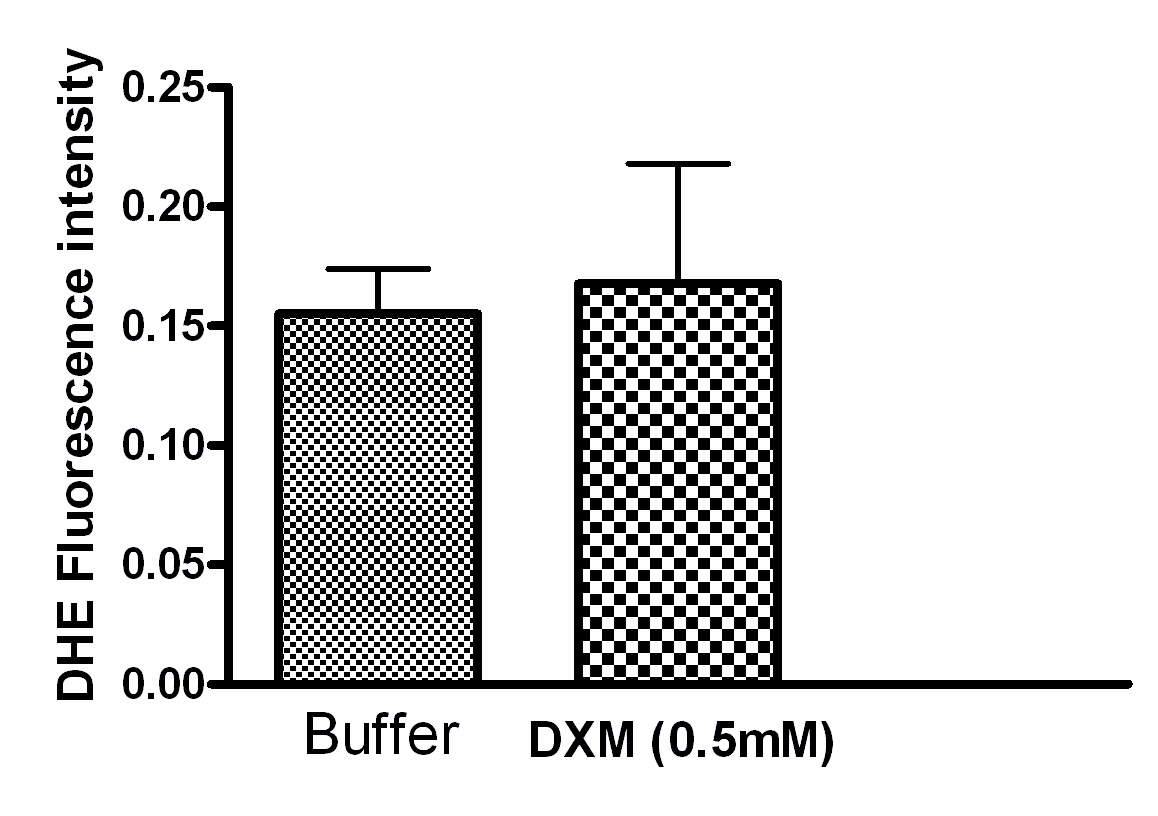
**

**Figure S8**
